# Induced systemic resistance against *Botrytis cinerea* by *Micromonospora* strains isolated from root nodules

**DOI:** 10.3389/fmicb.2015.00922

**Published:** 2015-09-02

**Authors:** Pilar Martínez-Hidalgo, Juan M. García, María J. Pozo

**Affiliations:** ^1^Department of Microbiology and Genetics, University of SalamancaSalamanca, Spain; ^2^Unidad Asociada USAL-CSIC “Interacción Planta-Microorganismo”Salamanca, Spain; ^3^Soil Microbiology and Symbiotic Systems, Estación Experimental del Zaidín, Consejo Superior de Investigaciones CientíficasGranada, Spain

**Keywords:** biocontrol, defense priming, induced systemic resistance, jasmonic acid, *Micromonospora*, PGPR, tomato

## Abstract

*Micromonospora* is a Gram positive bacterium that can be isolated from nitrogen fixing nodules from healthy leguminous plants, where they could be beneficial to the plant. Their plant growth promoting activity in legume and non-legume plants has been previously demonstrated. The present study explores the ability of *Micromonospora* strains to control fungal pathogens and to stimulate plant immunity. *Micromonospora* strains isolated from surface sterilized nodules of alfalfa showed *in vitro* antifungal activity against several pathogenic fungi. Moreover, root inoculation of tomato plants with these *Micromonospora* strains effectively reduced leaf infection by the fungal pathogen *Botrytis cinerea*, despite spatial separation between both microorganisms. This induced systemic resistance, confirmed in different tomato cultivars, is long lasting. Gene expression analyses evidenced that *Micromonospora* stimulates the plant capacity to activate defense mechanisms upon pathogen attack. The defensive response of tomato plants inoculated with *Micromonospora* spp. differs from that of non-inoculated plants, showing a stronger induction of jasmonate-regulated defenses when the plant is challenged with a pathogen. The hypothesis of jasmonates playing a key role in this defense priming effect was confirmed using defense-impaired tomato mutants, since the JA-deficient line *def1* was unable to display a long term induced resistance upon *Micromonospora* spp. inoculation. In conclusion, nodule isolated *Micromonospora* strains should be considered excellent candidates as biocontrol agents as they combine both direct antifungal activity against plant pathogens and the ability to prime plant immunity.

## Introduction

Most actinobacteria were considered to inhabit environments such as soil, rhizosphere, or lake sediments. However, it was later discovered that actinobacteria are closely associated with plants and they have been isolated from a great number of different plant genera, colonizing different parts of the plant (Coombs and Franco, 2003; Hasegawa et al., 2006; Qin et al., 2010; Velázquez et al., 2013). Actinobacteria have been described to promote plant growth and their beneficial effect has been reported previously in various plant species (El-Tarabily and Sivasithamparam, 2006; Franco et al., 2007).

Most legumes are engaged in a symbiotic relationship with nitrogen-fixing rhizobia hosted in root nodules. A common misconception was that nodules from leguminous plants were thought to be inhabited by only one type of microorganism, but it is now clear that they conform an ecosystem of their own: besides nodule forming bacteria, several other endophytes with PGP activities are found (Velázquez et al., 2013). Among other actinobacteria, *Micromonospora* strains have been found in a wide selection of leguminous plants, including *Medicago sativa*, the plant of choice for the isolation of *Micromonospora* spp. in our study. Previous studies (Martínez-Hidalgo et al., 2014b) showed that nodule isolated *Micromonospora* strains excel at plant growth promotion when inoculated in alfalfa. This effect was not due to biological nitrogen fixation although the nitrogen content was significantly higher than in control plants (Martínez-Hidalgo et al., 2014a).

A number of actinobacteria have been also described to reduce the negative effects of pathogens in plants, inhibiting pathogen growth via production of antibiotics, lytic enzymes or siderophores or inducing the plant defense mechanisms (Conn et al., 2008; Hirsch and Valdés, 2010; Verma et al., 2011) but very few have proven ability to promote plant growth, inhibit the growth of pathogens and also boost plant defensive capacity in plants of agronomic relevance.

Plants have developed mechanisms to detect potential aggressors and to coordinate the appropriate defense responses, including the production of toxic substances and lytic enzymes. Regulation of these responses is under phytohormone control, being salicylic acid (SA), jasmonic acid (JA) and ethylene among the major regulators (Pieterse et al., 2009). In general terms, JA coordinates responses effective against necrotrophic pathogens and chewing insects, while SA targets mainly biotrophic pathogens such as viruses, but intensive cross-talk among both pathways (generally antagonistic) allows the plant to shape the final immune response triggered against specific invaders (Pieterse et al., 2009). Upon appropriate stimulation plants can develop a state of enhanced defensive capacity, known as induced systemic resistance (ISR). Different soil beneficial microorganisms have been shown to trigger ISR in plants, usually relying on JA signaling (van Loon et al., 2006; Pozo and Azcón-Aguilar, 2007; Van Wees et al., 2008; Pieterse et al., 2014).

Induced resistance may result in direct activation of defense mechanisms – including increased basal levels of defense-related compounds, or the priming of the plant defensive capacity. In the latter, no major changes appear upon induction in the absence of a challenge, but a more efficient activation of defense mechanisms occurs upon attack. Thus, priming is a cost-effective way of increasing plant resistance (Conrath et al., 2006; Pastor et al., 2013).

The fungus *Botrytis cinerea* is a plant necrotrophic pathogen that colonizes senescent or dead plant tissues and causes gray mold in vegetables and softening in fruits. Its hyphae can penetrate plant tissues through wounds or natural openings and spread from previously colonized dead tissues into healthy ones. It has a broad host range of food crops, including tomato (*Solanum lycopersicum*), with the gray mold disease being responsible for substantial economical crop losses every year (Dean et al., 2012). Tomato is one of the most important crops in the world and it is considered an important model plant because, besides its economic importance, it display interesting features, a known genome and a considerable number of mutants and genomic tools available (Kimura and Sinha, 2008). Remarkably, *B. cinerea* is able to manipulate tomato defense regulatory pathways to promote fungal colonization and disease progression (El Oirdi et al., 2011; Rahman et al., 2012). *B. cinerea* control is usually achieved by cultural measures and application of broad spectrum fungicides.

The increasingly strict regulation on chemical pesticides and environmental and safety concerns, have evidenced the need of sustainable and safe solutions for crop protection. Thus, formulating bioinoculants with both growth and defense promotion for plants is a major goal in modern agriculture. *Micromonospora* strains have been isolated from leguminous root nodules and found to improve crop performance in alfalfa (Martínez-Hidalgo et al., 2014b). They are sporulating bacteria, a highly valued trait relevant for its use as bioinoculants, as it allows cultures to be stored for long periods of time without a significant loss in survival.

The aim of this study was to test the potential of *Micromonospora* strains isolated from alfalfa nodules as biocontrol agents exploring their antifungal properties and their ability to boost plant defense mechanisms using the agronomically relevant pathosystem tomato-*B. cinerea*. The potential of these strains for improving disease control in horticulture is discussed.

## Materials and Methods

### Bacterial and Fungal Cultures

All endophytic *Micromonospora* cultures were maintained in SA1 medium (Trujillo et al., 2005), while all pathogenic fungi were cultured in PDA at 24°C. *B. cinerea* to be used for plant bioassays was cultured similarly but on PDA supplemented with tomato leaves at 40 mg ml^-1^ (Vicedo et al., 2009). *Micromonospora* strains used in this study were isolated from *M. sativa* root nodules (**Table 1**).

**Table 1:** Strains of *Micromonospora* used in this study and comparison of gene *rrs* with already described species.

Strain (accession)	*rrs* identification	%	Source
AL4 (KF876221)	*Micromonospora viridifaciens* DSM 43909T (X92623)	99,52	Martínez-Hidalgo et al. (2014b)
AL16 (KF876222)	*Micromonospora saelicesensis* Lupac 09 (AJ783993)	99,65	Martínez-Hidalgo et al. (2014b)
AL20 (KF876223)	*Micromonospora chokoriensis* 2-19/6 (AB241454)	99,79	Martínez-Hidalgo et al. (2014b)
ALF1 (KF876224)	*Micromonospora humi* P0402 (GU459068)	99,51	Martínez-Hidalgo et al. (2014b)
ALF2 (KJ187181)	*Micromonospora narathiwatensis* BTG4-1 (AB193559)	98,76	This work
ALF4 (KF876225)	*Micromonospora coxensis* 2-30-b/28 (AB241455)	99,31	Martínez-Hidalgo et al. (2014b)
ALF7 (KF876233)	*Micromonospora saelicesensis* Lupac 09 (AJ783993)	99,86	Martínez-Hidalgo et al. (2014b)
ALFb5 (KF876226)	*Micromonospora saelicesensis* Lupac 09 (AJ783993)	99,86	Martínez-Hidalgo et al. (2014a)
ALFb7 (KF876227)	*Micromonospora saelicesensis* Lupac 09 (AJ783993)	99,58	Martínez-Hidalgo et al. (2014b)
ALFb1 (KF876228)	*Micromonospora echinospora* ATCC 15837 (U58532)	97,78	Martínez-Hidalgo et al. (2014b)
ALFpr18c (KF876230)	*Micromonospora tulbaghiae* TVU1 (EU196562)	99,93	Martínez-Hidalgo et al. (2014a)
ALFpr19a (KF876231)	*Micromonospora lupini* Lupac 14N (AJ783996)	99,31	Martínez-Hidalgo et al. (2014b)
ALFr4 (KF876234)	*Micromonospora cremea* CR30 (FN658654)	98,62	Martínez-Hidalgo et al. (2014b)

### *In vitro* Antagonistic Bioassays for Inhibition of Fungal Growth

Pathogens were selected based on their importance as plant pathogens in Spain. The species chosen were *Fusarium circinatum, Sclerotinia sclerotiorum, Rhizoctonia solani*, and *B. cinerea*.

Pathogens routinely grown in PDA cultures, were grown in petri dishes with SA1 medium (Trujillo et al., 2005) in order to confirm they could grow normally under these conditions. For the trial, the 13 selected *Micromonospora* strains (**Table 1**) were streaked in a thick line in the center of the plate and let grow for 7 days at 28°C. After this time, 1 cm plugs of PDA media containing actively growing fungi of two different species (*F. circinatum* and *R. solani* or *S. sclerotiorum* and *B. cinerea*) were located equidistant at both sides of the streak of *Micromonospora* sp. (Supplementary Figure S1) and incubated for 2 days at 24°C. Three replicates were performed for each fungus.

### Plant Material and *Micromonospora* Inoculation Procedures

Five tomato (*S. lycopersicum* L.) genotypes were used in our studies including the three cultivars ‘Roma,’ ‘Castlemart’ and ‘Moneymaker,’ and the following lines altered in defense signaling: the JA-deficient mutant *def1* (Howe et al., 1996; in background ‘Castlemart,’ gently provided by G. Howe, Michigan State University) and SA-impaired transgenic line *nahG* (Brading et al., 2000; in background ‘Moneymaker’ gently provided by J. Jones, John Innes Centre).

To test the capacity of *Micromonospora* spp. to ISR in plants against *B. cinerea*, tomato seeds were sterilized with sodium hypochlorite for 20 min and rinsed three times in sterile distilled water. Seeds were placed on sterile vermiculite, grown until the first true leaf appeared and then transplanted to pots with commercial substrate (Projar Seed Pro 5050, Spain). Plants were randomly distributed and grown in a greenhouse at 24/16°C with a 16/8 h photoperiod and 60% humidity, and watered three times a week with Long Ashton nutrient solution (Hewitt, 1966).

After transplantation, the tomato plants were used in two sets of experiments: to analyze long-term effect of *Micromonospora* spp. inoculation the plants were inoculated immediately after transplantation with each microbial strain. To analyze short-term effect of *Micromonospora* spp. inoculation, the plants were inoculated 24 h before the infection with the pathogen. The experiments were repeated at least twice, and for each experiment, five replicates per treatment were used.

Inoculation of tomato plants with the selected *Micromonospora* strains was performed with 1 ml of bacterial suspensions (10^9^ cells per ml) of each microbial strain grown on solid medium. The bacterial suspensions were carefully strewn in the soil near the roots of the seedling using a micropipette.

### Pathogen Bioassays

One month after transplantation, *B. cinerea* was inoculated either by adding plugs of the fungal culture to each leaflet or by spray on the leaflets of tomato plants with 10^6^ *B. cinerea* conidia (in whole plants or in detached leaves; Zhai et al., 2013). The extension of the disease was measured 48 and 72 h after the challenge.

#### *B. cinerea* Bioassays in Intact Plants

*Solanum lycopersicum* L. ‘Roma’ were grown in the greenhouse in pots of 1 L capacity, filled with commercial substrate (Compo Sana^®^ Universal potting soil, Compo Iberia S.L.) and inoculated with *Micromonospora* strains ALFb5 and ALFpr18c a month before inoculation with *B. cinerea*. Plugs of agar containing *B. cinerea* micellium were attached to seven leaflets in each plant and located in a humidity chamber kept at 20–23°C. 48 h after challenge with *B. cinerea*, the diameter of the necrotic lesions formed by the fungal hyphae in the leaflets was measured with the aid of a Vernier caliper (Martínez-Medina et al., 2013). Two measurements were taken for each lesion considering the biggest and the smallest diameter and the average between the two was calculated.

#### *B. cinerea* Bioassays on Detached Leaves

Tomato plants were grown as described above, and the inoculation with *Micromonospora* spp. was performed 30 days or 24 h before pathogen challenge.

In these experiments *B. cinerea* was applied to detached leaves. Two leaves of each plant were detached with a sharp blade, placed in plastic trays on wet paper and challenged with *B. cinerea* by applying 1 cm diameter plugs of PDA media containing actively growing mycelia of *B. cinerea* obtained from 3 weeks-old culture plates. The leaves were then placed in a humidity chamber and kept at 20–23°C with constant light. Disease damage level was scored after 72 h and three levels of damage were established, according to the severity of the symptoms: mild for necrosis extending 1–2 mm from the plug, moderate for necrosis ranging from 3 to 5 mm from the plug and severe for lesions that extended for more than one third of the leaflet (Supplementary Figure S2).

### Gene Expression Analysis

Quantitative analysis of defense related gene expression. To evaluate the effects of *Micromonospora* spp. inoculation on defense gene expression upon pathogen attack, *B. cinerea* was applied to detached leaves of tomato plants that had been inoculated with *Micromonospora* spp. at transplanting, 30 days before the challenge with the fungus. Two leaves of each plant were detached, placed in a humidity chamber as described above and challenged by spraying a spore suspension of *B. cinerea* on the leaf surface. This inoculation method ensures homogeneous and simultaneous contact of the leaf tissue with the pathogen, allowing a more precise quantification of changes in gene expression levels. Spores collected from 15 days-old cultures were incubated in Gambor’s B5 medium (Duchefa, Haarlem, The Netherlands) supplemented with 10 mM sucrose and 10 mM KH_2_PO_4_ for 2 h in the dark without shaking (Vicedo et al., 2009). The suspension was adjusted to 5 × 10^6^ spores ml^-1^. Controls were mock inoculated in a similar way. All leaves were kept under high humidity and harvested at 72 h.

Total RNA was extracted from tomato leaves taken from four different treatments: uninoculated and unchallenged control plants (Control), plants treated only with *Micromonospora* sp. (pr18c), plants challenged only with *B. cinerea* (*Botrytis*) and plants inoculated with *Micromonospora* sp. and then challenged with *B. cinerea* (pr18c+*Botrytis*).

The RNA was extracted following the Tri-Reagent (Sigma) protocol following the manufacturer’s procedure. Contaminating DNA was removed with RQ1 DNase (Promega), purified through a silica column using the NucleoSpin RNA Clean-up kit (Macherey- Nagel). cDNA was synthesized with 3 μg of purified total RNA using the iScript cDNA Synthesis kit (Bio-Rad) according to the manufacturer’s instructions. The conditions of RT-qPCR experiments and the relative quantification of specific mRNA levels was performed according to López-Ráez et al. (2010), using tomato gene specific primers previously described, coding for Pathogenesis related protein PR1a (ID M69247, *PR1a*; Martínez-Medina et al., 2013), Proteinase inhibitor II (ID K03291, *Pin II*; Uppalapati et al., 2005), Lipoxygenase A (ID U09026, *LoxA*; López-Ráez et al., 2010) and elongation factor 1α (ID X14449, *SlEF*; Rotenberg et al., 2006).

Expression values were normalized using the housekeeping gene *SlEF*, which encodes for the tomato elongation factor-1α. At least three independent biological replicates were analyzed per treatment, and each qPCR reaction was performed in duplicate.

### Detection of *Micromonospora* sp. in Plant Tissues

Presence of *Micromonospora* in plant roots and shoots was assessed by amplification of *Micromonospora* DNA in the samples by PCR. Primers to amplify the *gyrB* gene, which encodes for the subunit B of DNA gyrase, were designed in our laboratory on the basis of available sequences of this gene in the *Micromonospora* genus. The primer sequences are: F: TCGACGGCAAGGCGTACG and R: CGCAGCTTCTCSATGTCG. Genomic DNA of root or leaf samples was extracted using NucleoSpin^®^ Plant II columns (Macherey Nagel, Duren, Germany). DNA quality and PCR performance were confirmed in all samples by amplification of the tomato *SlEF* gene. Bacterial DNA obtained from a pure *Micromonospora* culture was used as a positive control for *gyrB* amplification. *gyrB* amplification conditions were as follows: 9 min at 95°C, 35 cycles of 1 min at 94°C, 1 min at 62°C and 2 min at 72°C, followed by 7 min final extension at 72°C. PCR products were electrophoresed in 1% agarose gels containing ethidium bromide (0.5 μg/ml) using modified Tris-Acetate EDTA buffer (Millipore, Cork, Ireland).

### Statistical Analysis

The statistical analysis of data was performed using SPSS software, version 21 (SPSS, Inc., Chicago, IL, USA). The data on lesion diameter in tomato leaves was processed with one-way analysis of variance (ANOVA). The statistical significance of the results was determined using Fisher’s LSD test (*P* < 0.05).

Association between severity of leaf fungal damage and inoculation treatments was examined using Chi-square tests, followed by *z*-tests to compare the inoculation groups at each damage level. A criterion of *P* < 0.05 for statistical significance was used for all analyses and *P*-values were corrected for multiple tests using the Bonferroni correction.

## Results

### *Micromonospora* Strains Inhibit the Growth of Fungal Plant Pathogens *In Vitro*

The strains under study were isolated from alfalfa root nodules and have been found to have great genetic diversity according to the *rrs* gene sequences (**Table 1**).

Selected *Micromonospora* isolates were tested for their ability to inhibit the growth of the damaging fungal pathogens *F. circinatum, S. sclerotiorum, R. solani*, and *B. cinerea* (Supplementary Figure S1).

All of the 13 isolates assayed inhibited the growth of one or more of the selected pathogenic fungi (**Table 2**). Only two strains of *Micromonospora* were capable of inhibiting growth of *F. circinatum* (ALF4 and ALFb7), five strains (AL4, AL16, AL20, ALFpr18c y ALFr4) inhibited *S. sclerotinum*, seven strains were inhibitory to *R. solani* (AL20, ALF1, ALF2, ALFb5, ALFb7, ALFpr18c y ALFpr19a) and 10 strains inhibited *B. cinerea* (AL4, AL20, ALF1, ALF2, ALF7, ALFb1, ALFb5, ALFpr18c, ALFpr19a y ALFpr4). The results suggest the potential of these strains to control fungal diseases through direct effects on the pathogen.

**Table 2:** Antimicrobial activity of selected strains of *Micromonospora* against four selected plant pathogenic fungi.

Strains	AL4	AL16	AL20	ALF1	ALF2	ALF4	ALF7	ALFb1	ALFb5	ALFb7	ALFpr18c	ALFpr19a	ALFr4
*Fusarium circinatum*	-	-	-	-	-	+	-	-	-	+	-	-	-
*Sclerotinia sclerotiorum*	+	+	+	-	-	-	-	-	-	-	+	-	+
*Rhizoctonia solani*	-	-	+	+	+	-	-	-	+	+	+	+	-
*Botrytis cinerea*	+	-	+	+	+	-	+	+	+	-	+	+	+

*Micromonospora* strains ALFb5 and ALFpr18c, were selected for *in planta* studies as they inhibited a large number of fungal pathogens (three for ALFpr18c and two for ALFb5) and were previously shown to efficiently promote plant growth (Martínez-Hidalgo et al., 2014b).

### *Micromonospora* sp. Induce Systemic Resistance against *B. cinerea* in Tomato

The two selected *Micromonospora* strains (ALFpr18c and ALFb5) were tested for its efficiency to increase tomato resistance against *B. cinerea*.

**FIGURE 1:**
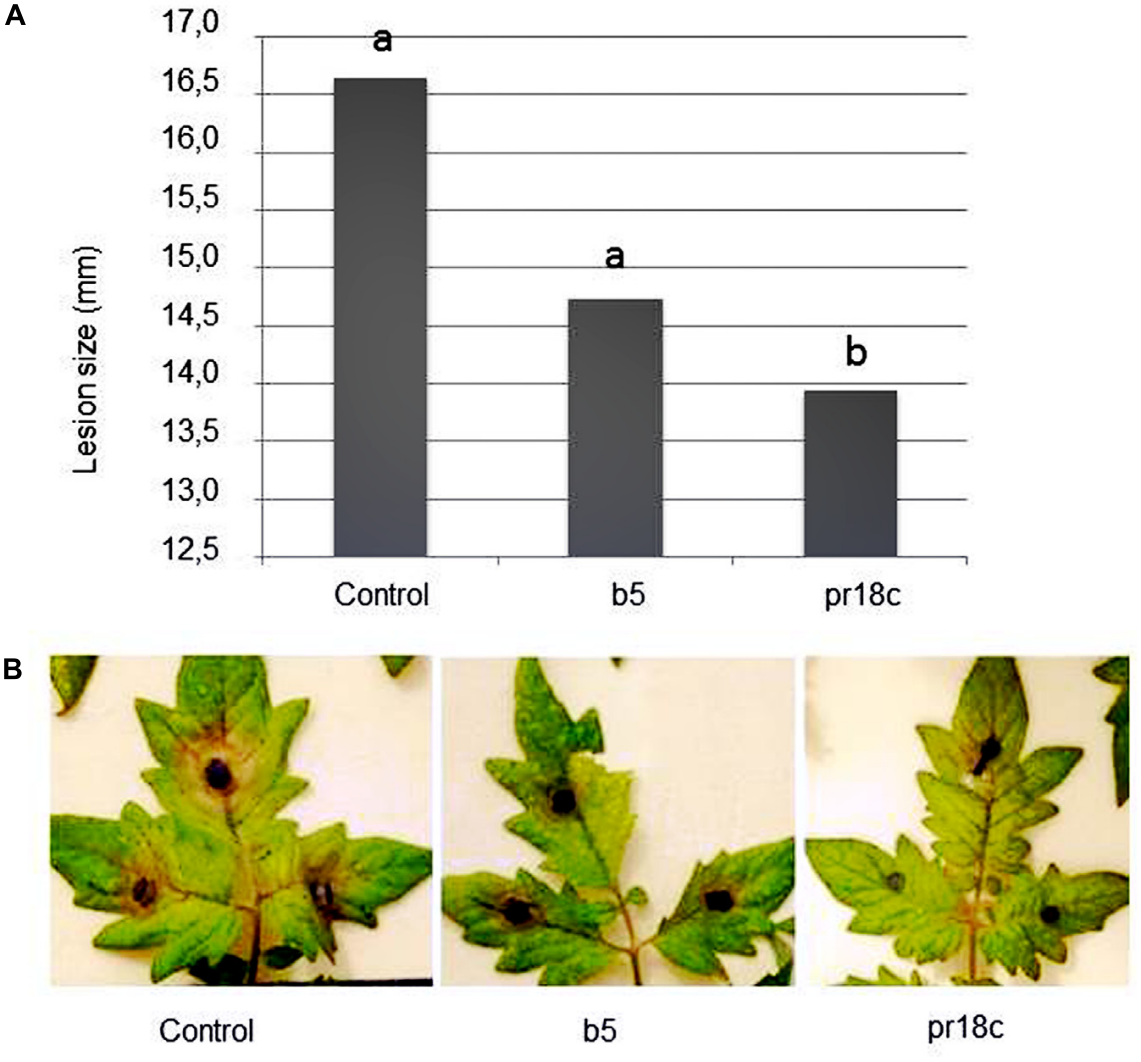
**Effect of root inoculation with selected *Micromonospora* strains (ALFb5 and ALFpr18c) on tomato resistance against *Botrytis cinerea*. (A)** Symptom severity in leaves of tomato plants upon challenge with *B. cinerea* was determined by measuring the diameter of the necrotic lesions 42 h post-pathogen inoculation. Plants were grown in the absence of *Micromonospora* (Control) or inoculated at transplanting with *Micromonospora* strain ALFpr18c (pr18c) or strain ALF b5 (b5). Data not sharing a common letter are significantly different (Fisher’s protected LSD test at *P* ≤ 0.05). **(B)** Examples of *B. cinerea* symptom development in leaves of *Micromonospora* spp. inoculated (b5 or PR18c) or non-inoculated (control) plants.

*S. lycopersicum* L. ‘Roma’ plants were root inoculated with *Micromonospora* strains at transplanting or 24 h prior challenge with *B. cinerea*, as described in materials and methods. Plants treated with any of the two *Micromonospora* strains looked healthier than control plants, even though only plants treated with *Micromonospora* sp. ALFpr18c showed less disease symptoms at the long term (**Figure 1**).

Remarkably, this significant reduction on the severity of symptoms caused by the pathogen was also evidenced even when pathogen inoculation was performed on detached leaves in a different experiment. As *Micromonospora* sp. ALFpr18c was consistently the most efficient strain in reducing disease severity, it was selected for the follow up studies.

**FIGURE 2:**
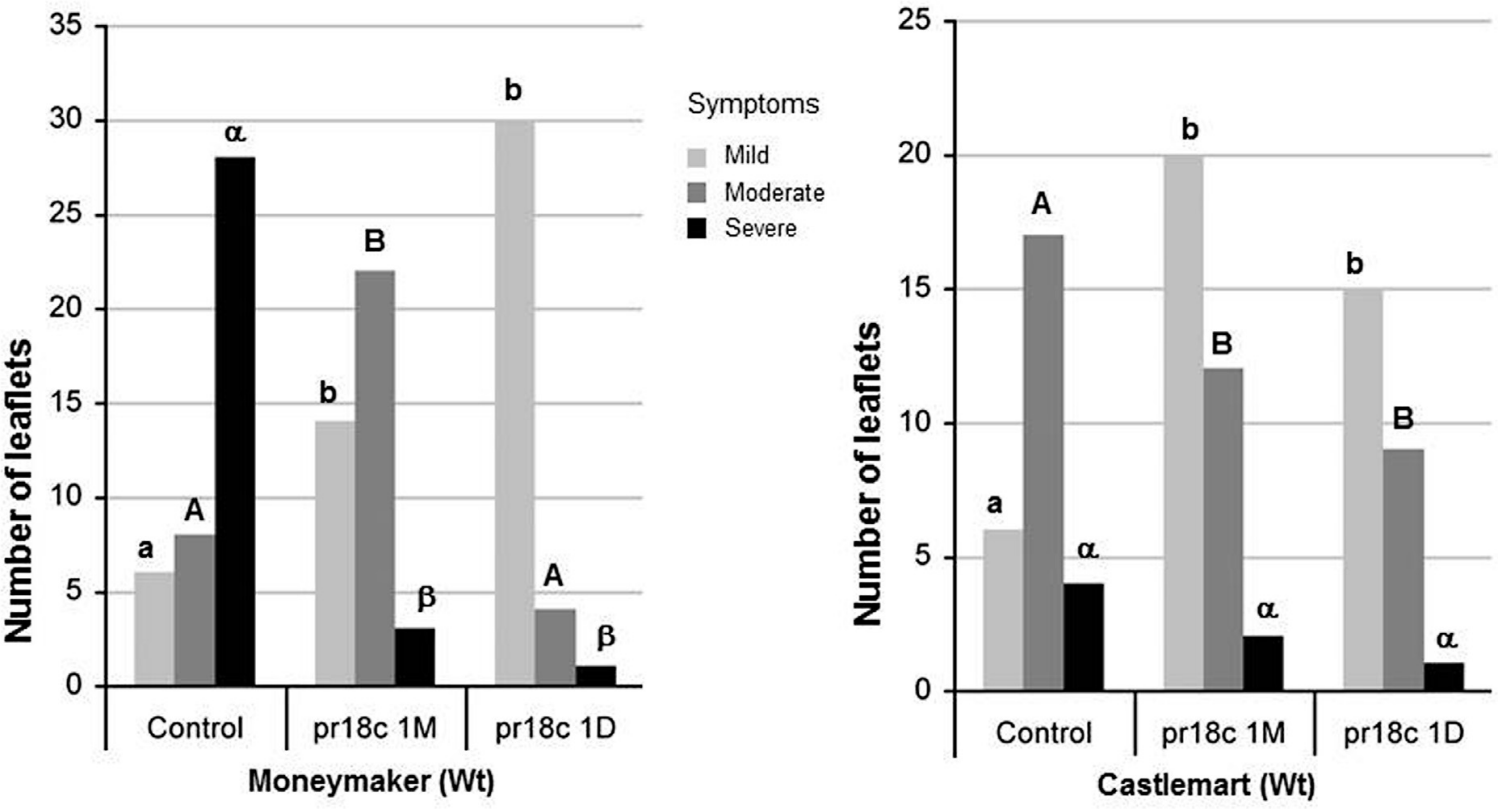
**Effect of *Micromonospora* ALFpr18c root inoculation on *B. cinerea* disease development in different tomato cultivars (*Solanum lycopersicom* L. ‘Moneymaker’ and ‘Castlemart’).** Necrosis severity caused by *Botrytis* was scored using a three levels disease scale: mild, moderate and severe, as shown in Supplementary Figure S2. The number of leaflets within each category is shown. For each tomato cultivar, disease damage level was compared between the control and the bacterial treatments. Control, plants not inoculated with *Micromonospora*; pr18c 1M and pr18c 1D, plants inoculated with *Micromonospora* sp. pr18c 1 month or 1 day before challenge with *B. cinerea*, respectively. Bars not sharing a common letter (lowercase for mild, uppercase for moderate and greek for severe symptoms) are significantly different using Bonferroni corrected Chi-square tests, followed by *z*-tests (*P* ≤ 0.05).

We extended our analysis to other tomato cultivars to find out if the protection by *Micromonospora* sp. ALFpr18c was a consistent effect for tomato and not a cultivar-specific response, choosing two well-characterized cultivars with defense impaired mutants available: ‘Castlemart’ and ‘Moneymaker.’ The sensitivity to *B. cinerea* of the two tomato cultivars differ significantly, being ‘Castlemart’ less severely affected than ‘Moneymaker’ (*X*^2^ = 18,871, *P* = 0.001; **Figure 2**). Remarkably, inoculation with *Micromonospora* spp. resulted in a significant reduction of the disease symptoms in both cultivars. The ISR by ALFpr18c against *B. cinerea* was effective regardless the timing of the inoculation of the bacteria, 30 days or 24 h before the challenge with the pathogen (**Figure 2**). *Micromonospora* sp. protected plants challenged with *B. cinerea* by reducing the severity of damage caused by the pathogen, as it was shown by a significant decrease in the number of leaflets with the highest level of damage (**Figure 2**). Indeed, there was a statistically significant association between levels of leaf fungal damage and inoculation treatments for ‘Castlemart’ (*X*^2^ = 8,374, *P* = 0.015) and for ‘Moneymaker’ (*X*^2^ = 29,824, *P* = 0.001).

The spatial separation between both microorganisms (*Micromonospora* was inoculated in the soil, close to the roots, and the pathogen in the leaves) suggest that the protection is related to the activation of plant defense mechanisms. However, to rule out a possible direct antifungal activity of the bacteria through an eventual colonization of the leaf tissue in the long term treatments, we evaluated the presence of the bacteria in roots and leaves of inoculated and non-inoculated tomato plants using *Micromonospora* gene *gyrB* specific primers. *GyrB* amplification, and therefore, the presence of *Micromonospora* sp. ALFpr18c was evidenced in the roots of the plants inoculated at transplanting, 30 days before harvesting, confirming effective root colonization by *Micromonospora*. However, no bacterial gene amplification was detected in the leaves of the same plants, confirming the bacteria localization in the roots and the physical separation of the resistance inducer and the pathogen (Supplementary Figure S3).

### *Micromonospora* sp. Induced Resistance is Related to Priming of Jasmonate-Regulated Responses

**FIGURE 3:**
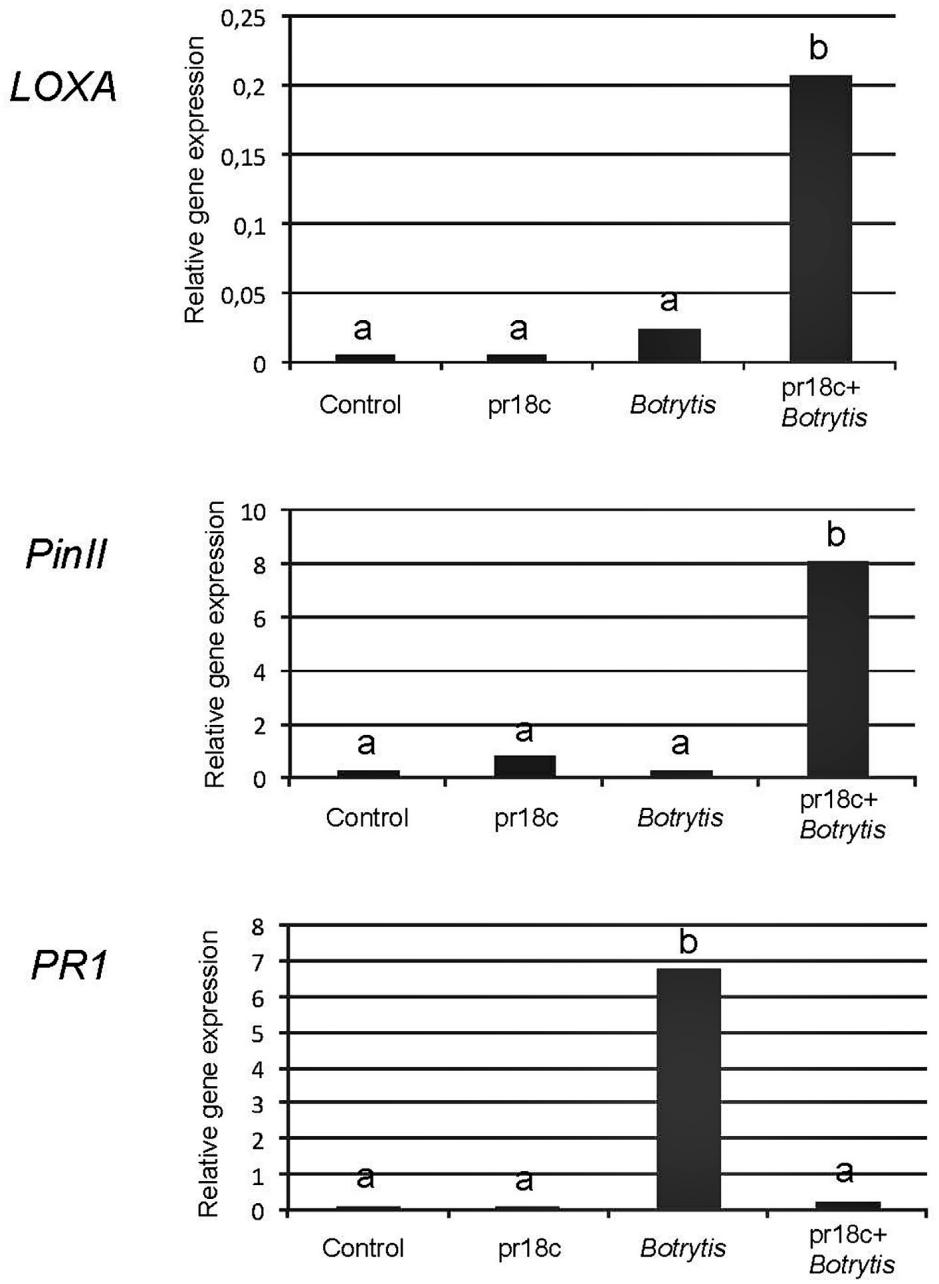
**Expression levels of the defense related genes *PR1*, *LoxA*, and *PinII* in leaves of tomato plants.** Relative expression levels for *PR1*, *LoxA*, and *PinII* were determined by qPCR and normalized against the constitutive *SlEF* (tomato elongation factor α). Control: uninoculated, unchallenged plants; pr18c: plants inoculated with *Micromonospora* strain ALFpr18c; *Botrytis*: uninoculated plants challenged with *B. cinerea*; pr18c+*Botrytis*: plants previously inoculated with *Micromonospora* strain ALFpr18c and challenged with *B. cinerea*. *Micromonospora* inoculation was performed at transplanting, a month before infection with *Botrytis*. Data not sharing a common letter are significantly different according to Fisher’s protected LSD test at *P* ≥ 0.05.

To understand the mechanism behind this long term induced resistance, and to determine what defense signaling pathways were involved, we compared the response against *B. cinerea* in plants inoculated or not with the strain *Micromonospora* sp. ALFpr18c 30 days before the challenge with the pathogen. Plant defense responses to *Botrytis* are known to depend on the interplay between SA and JA dependent responses, and those regulated by JA have been proposed as the major players in resistance (El Oirdi et al., 2011). Accordingly, we monitored the expression levels of well-characterized marker genes for both signaling pathways in leaves from the different treatments. Non-challenged plants showed no differences in the expression levels of any of the marker genes analyzed regardless of the presence of *Micromonospora* sp. (**Figure 3**). In contrast, important differences were found in the transcription levels of marker genes from both pathways between non-inoculated plants and plants preinoculated with *Micromonospora* spp. upon challenge with *B. cinerea* (**Figure 3**).

The *PR1* gene, encoding for an acidic form of the Pathogenesis Related Protein 1, is well-recognized as a marker of SA dependent responses. The expression of *PR1* was strongly induced in control plants upon challenge with the pathogen (sevenfold), but the increase upon *B. cinerea* challenge was much lower in plants previously inoculated with *Micromonospora* sp.

Regarding the JA regulated signaling pathway, the expression of the marker genes *PinII*, encoding for Proteinase inhibitor II, known to be involved in the plant resistance to *B. cinerea*, and *LoxA*, coding for a JA-inducible lipoxygenase involved in the biosynthesis of oxylipins was monitored. Transcript levels of both *PinII* and *LoxA* genes were significantly higher in plants inoculated with *Micromonospora* sp. and then challenged with *B. cinerea* than in the rest of treatments (**Figure 3**). Remarkably, control plants without *Micromonospora* sp. but challenged with *B. cinerea* showed very low levels of expression of both JA-marker genes, indicating a poor activation of the efficient (JA regulated) defense mechanisms in the absence of *Micromonospora* ALFpr18c.

### *Micromonospora* sp. Induced Resistance against *B. cinerea* is Compromised in Defense-Deficient Tomato Mutants

**FIGURE 4:**
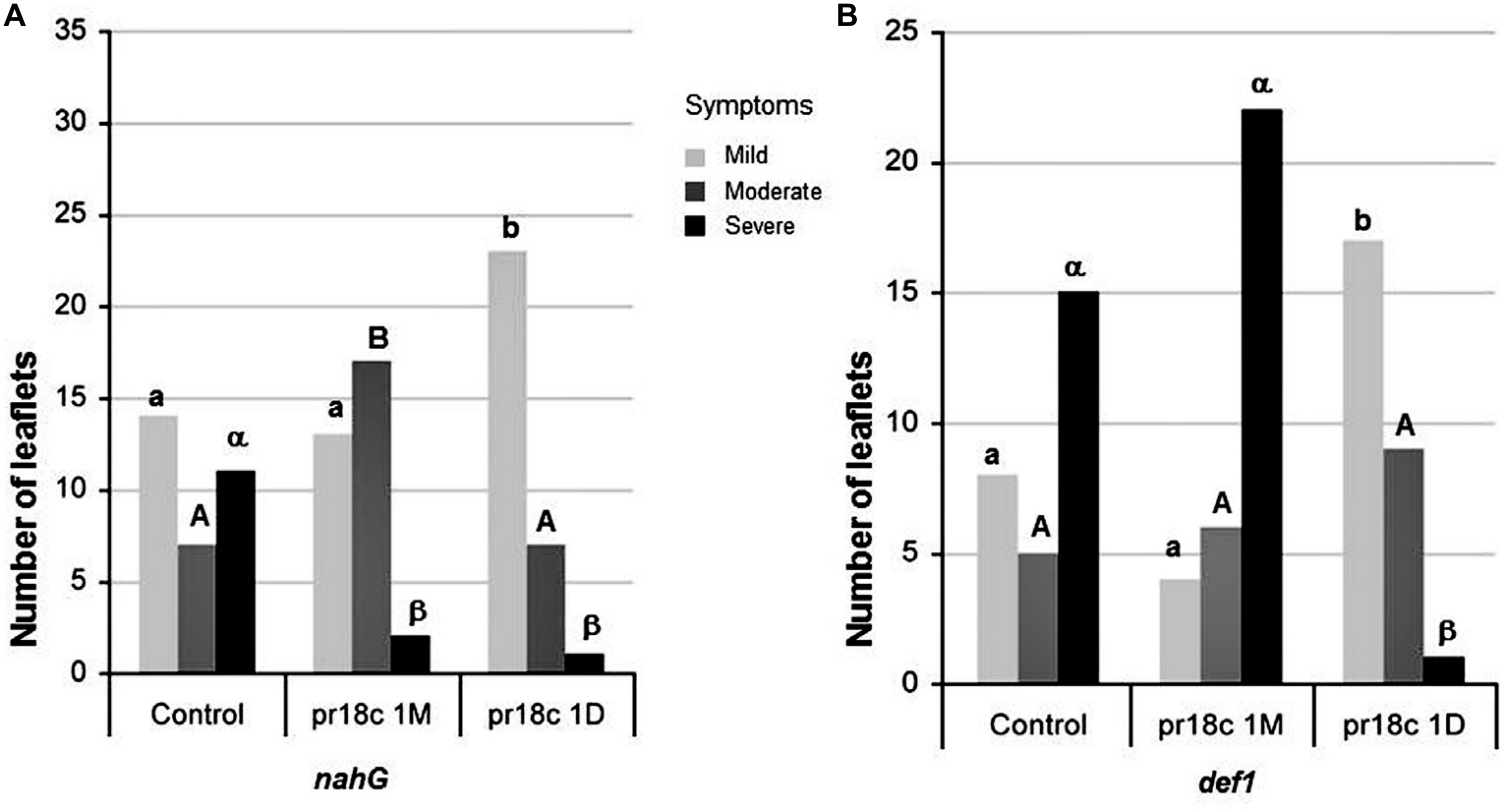
**Effect of *Micromonospora* ALFpr18c root inoculation on disease development caused by *B. cinerea* in different defense deficient tomato lines. (A)** SA-deficient *nahG* and **(B)** JA-deficient *def 1* plants. Necrosis severity caused by *B. cinerea* was scored using a three levels disease scale: mild, moderate and severe, as shown in Supplementary Figure S2. Control, plants not inoculated with *Micromonospora*; pr18c 1M and pr18c 1D, plants inoculated with *Micromonospora* sp. ALFpr18c 1 month or 1 day before challenge with *B. cinerea*, respectively. Within each genotype, bars not sharing a common letter (lowercase for mild, uppercase for moderate and greek for severe symptoms) are significantly different using Bonferroni corrected Chi-square tests, followed by *z*-tests (*P* ≤ 0.05).

The ability of *Micromonospora* sp. ALFpr18c to induce resistance against *B. cinerea* was analyzed in tomato plants impaired in JA or SA signaling (**Figure 4**). Tomato plants were challenged with *B. cinerea* 30 days after root inoculation with *Micromonospora* sp., as described in Section “Materials and Methods.”

To address the possible role of the SA regulated defense pathway in the *Micromonospora* ISR, *nahG* plants and their corresponding wild type (‘Moneymaker’) were used. *nahG* plants, unable to accumulate SA, were significantly less susceptible to *B. cinerea* than their corresponding wild type (*X*^2^ = 9,499, *P* = 0.009). Thus, SA appears to have a negative role in disease resistance, in agreement with recent reports showing that *B. cinerea* manipulate SA signaling to promote infection and disease progression (El Oirdi et al., 2011).

When studying the effect of ALFpr18c on *nahG* mutant, there is a statistically significant association between levels of leaf fungal damage and inoculation treatments: (*X*^2^ = 10,434, *P* = 0.005); *Micromonospora* sp. inoculated *nahG* plants showed a significant reduction on the severity of symptoms caused by the pathogen, as evidenced by the decrease in the percentage of leaves with higher severity symptoms (**Figure 4**). No significant differences were found (*X*^2^ = 0,190, *P* = 0.909) when comparing ALFpr18c-inoculated *nahG* or wild type (Moneymaker) plants, confirming that *Micromonospora* induced resistance was not impaired in the SA deficient mutant.

To analize the relevance of JA signaling on *Micromonospora* ISR, *def1* mutants, deficient in JA regulated responses and their corresponding wild type cultivar (‘Castlemart’) were used. Differences in the basal resistance of *def1* and its parental line (‘Castlemart’) were clear, being *def1* plants significantly more susceptible to *B. cinerea* than the corresponding wild type cultivar (*X*^2^ = 13,186, *P* = 0.001; **Figure 4**), supporting the key role of JA in resistance to *B. cinerea*. When analyzing the effect of ALFpr18c on *def1* mutant, there is not a statistically significant association between levels of leaf fungal damage and inoculation treatments: (*X*^2^ = 2,493, *P* = 0.288). Accordingly, plant protection against the pathogen by the strain ALFpr18c is lost in the JA deficient mutant.

Taken all together, our results show that JA has a positive role in tomato resistance against *B. cinerea*, and these responses are effectively primed by *Micromonospora* sp.

We also tested the efficacy of the short term induction of resistance, comparing disease severity when *Micromonospora* ALFpr18c inoculation was performed 24 h before the challenge. Plants treated with ALFpr18c showed less severe lesions than control plants regardless the tomato line tested, even in the case of the JA compromised mutant *def1*, unable to maintain long term ISR (**Figure 4**).

## Discussion

In the context of the new agricultural sustainability directives that have been outlined by the European Union, the search for effective bioinoculants is a major goal in agronomic research. The *Micromonospora* strains presented here, isolated from alfalfa root nodules showed very good plant growth promoting activity (Martínez-Hidalgo et al., 2014a). Plant growth promotion is often achieved by both improved plant nutrition (Vessey, 2003; Velázquez et al., 2013) and enhanced stress tolerance, for example, reducing disease damages. In this study we evaluated the potential of *Micromonospora* strains for biological control of pathogens. We evaluate the two different ways by which *Micromonospora* spp. could act as biocontrol agents, by testing their direct inhibitory activity against plant pathogens, and their ability to boost plant defense mechanisms.

Most of the *Micromonospora* strains tested showed a clear inhibitory effect on multiple plant pathogenic fungi in *in vitro* antagonistic assays (**Table 2**). Production of secondary metabolites is common in *Actinobacteria*, many of them with antibiotic effect (El-Tarabily et al., 1997; Gadelhak et al., 2005; Hirsch and Valdés, 2010). Production of antimicrobial substances by biocontrol agents is associated to plant protection by selective inhibition of fitopathogenic fungi, thus avoiding crop losses linked to diseases. Root diseases caused by soil pathogens are a major problem in agriculture and antagonism in the rhizosphere is an effective tool to decrease their incidence and damage. Inoculation with *Micromonospora* spp. may contribute to this antagonistic effect. The metabolic versatility of *Micromonospora* spp. is very high, they are able to produce multiple metabolites, such as antibiotics (Weinstein et al., 1966), antitumorals (Igarashi et al., 2007) or lytic enzymes, like chitinases, or proteases that could inhibit germination of *B. cinerea* spores or suppress fungal growth (Frankowski et al., 2001; Kamensky et al., 2003). Further studies will be needed to discover the metabolites or enzymes responsible for the observed inhibitory effects of our selected strains.

Besides a direct antimicrobial effect, that can contribute to reduce pathogens propagation in the soil, *Micromonospora* sp. ALFpr18c is able to stimulate plant defenses and ISR against foliar pathogens. Here we show that root inoculation with *Micromonospora* sp. enhanced disease resistance to the foliar pathogen *B. cinerea*. For every tomato cultivar tested in the different trials (‘Roma,’ ‘Moneymaker’ and ‘Castlemart’), the extent and severity of the symptoms caused by *B. cinerea* in plants pretreated with *Micromonospora* sp. was significantly lower than in untreated controls.

*Micromonospora* sp. inoculation was performed in the soil, near the root system, while *B. cinerea* was directly applied to leaves, so that there was no contact between both microorganisms. Absence of bacteria on the leaves was confirmed by DNA amplification analysis. Thus, the direct antagonism of the bacteria on the fungus in our experimental conditions is very unlikely. The protection observed was, therefore, likely related to an effect on the plant defense mechanisms, further confirmed by the lack of *Micromonospora* triggered ISR in the *def1* defense deficient mutant lines.

The two *Micromonospora* strains tested in plant bioassays, ALFpr18c and ALFb5, reduced disease progression when inoculated 24 h before challenging the plants with *B. cinerea*. However, only ALFpr18c was able to induce long term systemic resistance, since inoculation at transplanting -30 days before challenge- with these bacteria, but not with ALFb5, did reduce disease severity significantly. For this reason, ALFpr18c was selected as an effective inducer of durable resistance and used to uncover the mechanisms underlying such effect.

Plant defense responses to *B. cinerea* are coordinated by the interplay of the JA and SA regulated signaling pathways (El Oirdi et al., 2011), the two major branches of defense related signaling. Quantitative analysis of transcript levels of marker genes for both pathways and the use of tomato lines defective in the signaling pathways allow to determine which pathways are activated in the different treatments, as previously described for other microbial inducers of plant resistance (Martínez-Medina et al., 2013). In the absence of pathogen attack, the bacteria do not have a clear impact on these major defense signaling pathways in the leaves (**Figure 3**). In contrast, the pathogen *B. cinerea* triggers a strong induction of the SA pathway, as previously described (El Oirdi et al., 2011). It has been proven that *B. cinerea* is sensitive to JA regulated responses, but not to those regulated by SA. Since SA and JA pathways are mutually antagonistic, the pathogen manipulate SA signaling to downregulate JA dependent responses to promote disease and necrosis spreading (El Oirdi et al., 2011; Rahman et al., 2012). The negative role of SA in tomato resistance against *B. cinerea* is here confirmed by the fact that the SA impaired line *nahG* was significantly less susceptible to *B. cinerea* than its corresponding wild type background (**Figure 4**), so SA pathway impairment renders the plant less susceptible to the fungus. Remarkably, the plants previously treated with the bacteria showed a minimal induction of the SA pathway upon challenge, thus the bacteria prevented the SA response triggered by the pathogen. In contrast, JA regulated responses were strongly upregulated in the preinoculated plants (**Figure 3**), particularly, *Pin II* that encodes for a proteinase inhibitor with strong inhibitory effects on *B. cinerea* (El Oirdi et al., 2011). As *Micromonospora* sp. itself does not induce a response from the plant, but this increase in defense was only evident upon challenge, the results support that *Micromonospora* sp. ISR against *B. cinerea* through priming of JA regulated defense responses. Priming is a cost efficient way of inducing resistance since defenses are only boosted upon pathogen attack, but they remain in basal levels in the absence of pathogen pressure (Conrath et al., 2006). Other beneficial microorganisms prime JA dependent responses, appearing as a common mechanism for efficient resource management in beneficial plant-microbe interactions (Van Wees et al., 2008; Selosse et al., 2014). It has been postulated that during initial stages of the interaction, beneficial soil microorganisms are perceived as potential aggressors by the plants, thus triggering some general defense responses. Later on, the beneficial bacteria deal with the plant immune system modulating plant defenses to achieve successful colonization, and as result, defenses may remain under “alert,” or primed (Zamioudis and Pieterse, 2012).

The analysis of the induction of resistance in tomato lines altered in the JA and SA signaling pathways further support this notion. The different lines showed a decrease in the severity of *B. cinerea* symptoms when inoculated with *Micromonospora* sp. Plants that were inoculated 24 h before the challenge with the pathogen show a reduction in disease severity in all tomato lines, including the JA and SA signaling deficient lines *nahG* and *def1*. Thus, this protection is probably due to an early general defense response to the bacteria that affect *B. cinerea*, but differs from the long term protective effects. Differences in the mechanisms underlying early and late responses associated to priming have been described, with early responses associated to the accumulation of reactive oxygen species and cell wall reinforcements, and late responses being under phytohormone control (Pastor et al., 2013), and a similar distinction may be applying here. In fact, the early unspecific defense response from the plant upon colonization by beneficial bacteria seems frequent. Even *Rhizobium*, known for establishing a very well-coordinated symbiosis with legumes, triggers a peak of defense pathway activation during the early stages of the interaction (Santos et al., 2001; Soto et al., 2006).

When *Micromonospora* sp. was inoculated at transplanting, 30 days before the challenge, disease severity was significantly reduced in the different tomato cultivars tested lines. However, the induction of resistance was completely lost in the JA deficient mutant *def1*, that was even more severely affected when inoculated with the bacteria.

In summary, the transcriptional analysis and the genetic approach with tomato deficient lines evidenced that the durable systemic resistance induced by *Micromonospora* sp. ALFpr18c is based on priming of JA regulated defense responses. JA dependent defenses, although mainly effective against necrotrophs, may also affect hemibiotrophs and even biotrophs (Pozo et al., 2005), so the spectrum of efficiency of *Micromonospora* sp. induced resistance deserves further exploration.

In this article, we provided evidences of the potential of *Micromonospora* strains as biocontrol agents for long lasting crop protection against phytopathogenic fungi. The priming activity of *Micromonospora* spp. is sustained in time -more than a month in our experiments- without significantly reducing its effectiveness, therefore inoculation with these bacteria could be performed at the time of sowing without a reduction in the effectiveness of the protection over time. These results, together with the direct antifungal potential evidenced for these strains, their proven role as plant probiotic bacteria and their sporulation capacity makes bacteria from the genus *Micromonospora* a very promising source of multifunctional bioinoculants.

## Conflict of Interest Statement

The authors declare that the research was conducted in the absence of any commercial or financial relationships that could be construed as a potential conflict of interest.
