# Supplementary material for: Induced systemic resistance against Botrytis cinerea by Micromonospora strains isolated from root nodules
Source: Front Microbiol. 2015 Sep 2;6:922. doi: 10.3389/fmicb.2015.00922 (PMC4556977; doi:10.3389/fmicb.2015.00922)
Supplement: Supplementary file 1 [file Supplementary_Material.PDF]

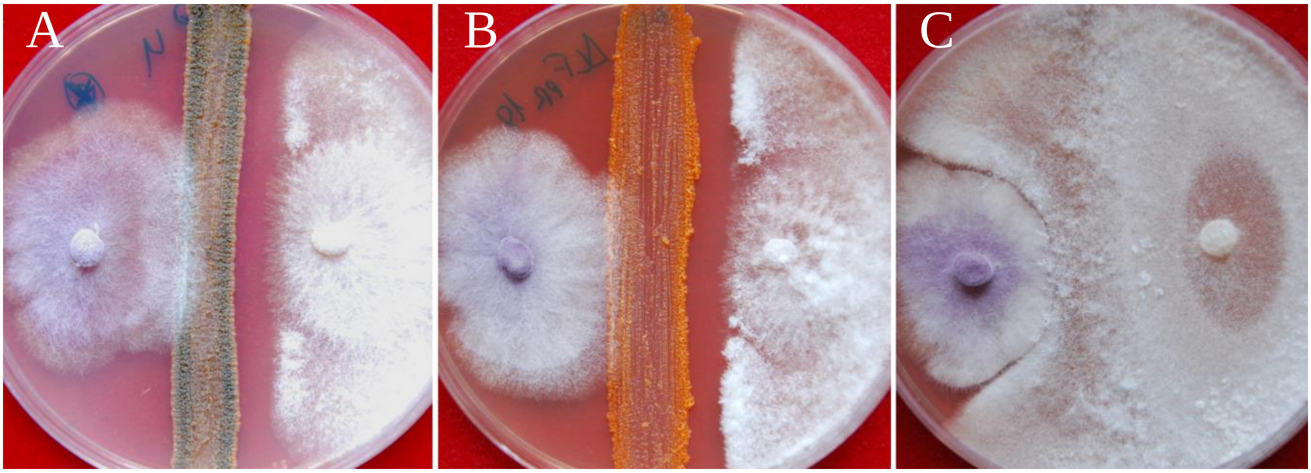

Figure 1S. *In vitro* antifungal activity assay of *Micromonospora* strains on fungal plant pathogens. A and B: Strains ALFpr19a and ALF4 inhibit growth of the fungal pathogen *Sclerotinia sclerotiorum* but do not affect the growth of *Fusarium circinatum* (C).

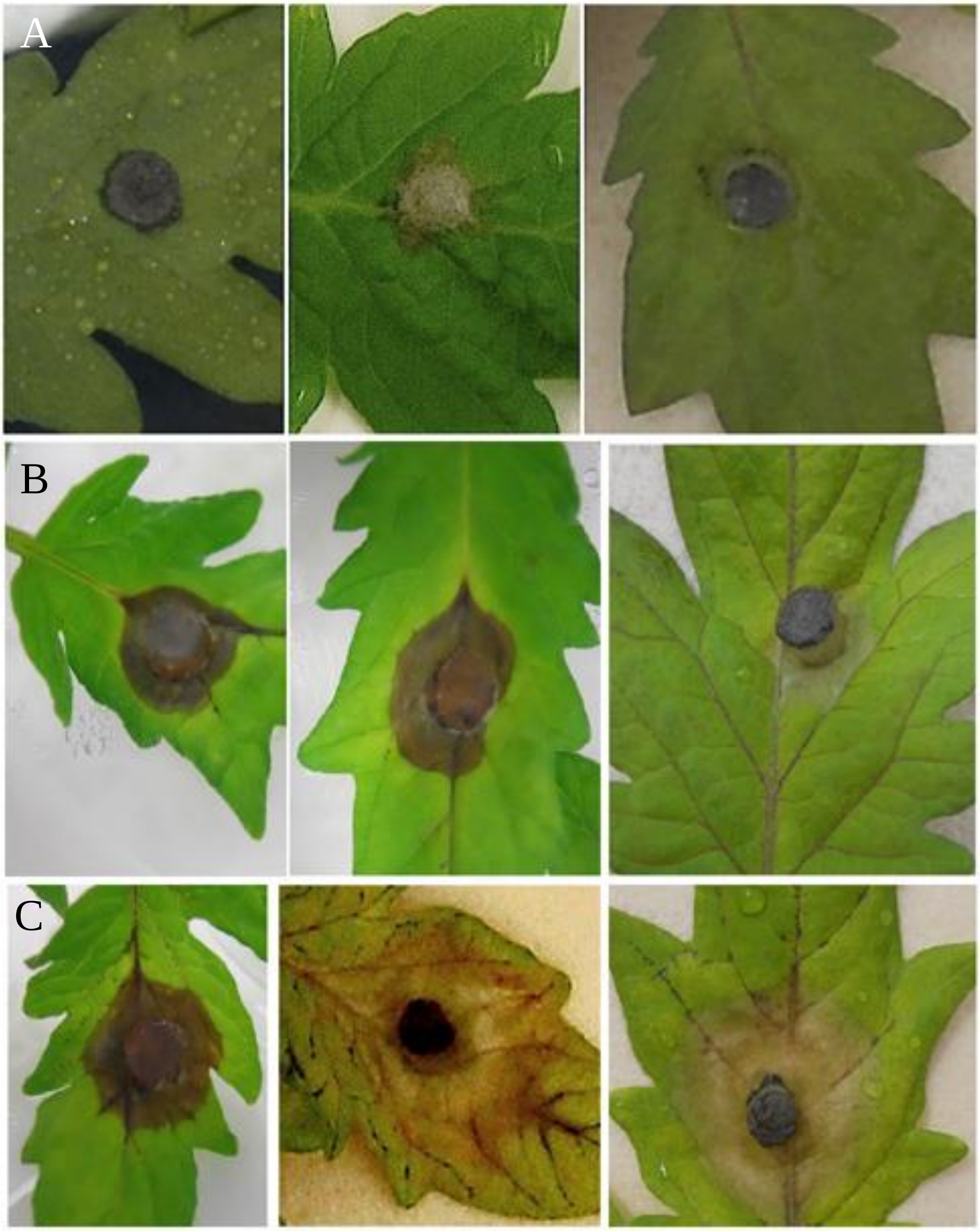

Figure 2S. Examples of disease development upon inoculation of leaves with the necrotrophic pathogen *Botrytis cinera*. Three levels of damage were established for symptoms severity caused by *Botrytis* in detached tomato leaves: A. Mild, B. Moderate, C. Severe

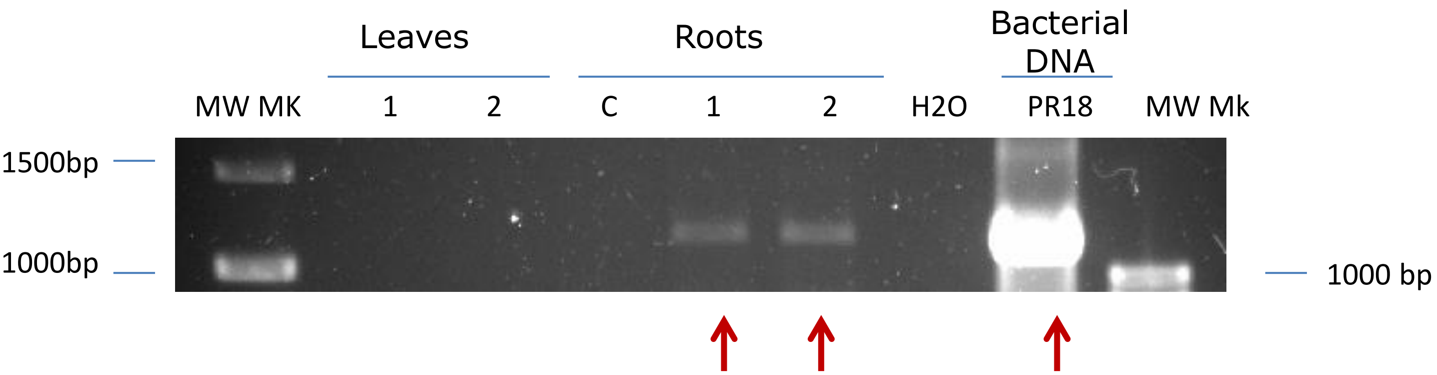

Figure S3. Agarose gel electrophoresis of PCR products from *Micromonospora gyrB* gene amplicons. 1 and 2: DNA samples from leaves and roots of the plants inoculated at transplanting with *Micromonospora* strain ALFpr18c, used for bioprotection and gene expression analysis. As negative control, samples from roots of non inoculated plants were used (line c), and DNA from a pure culture of *Micromonospora* ALFpr18c was included as positive control (line pr18). MW Mk: Molecular weight markers.
